# Developing a proxy version of the Adult social care outcome toolkit (ASCOT)

**DOI:** 10.1186/s12955-017-0682-0

**Published:** 2017-05-19

**Authors:** Stacey Rand, James Caiels, Grace Collins, Julien Forder

**Affiliations:** Quality and Outcomes of person-centred care policy Research Unit (QORU), University of Kent, Cornwallis Building, University of Kent, Canterbury, CT2 7NF UK

**Keywords:** Quality of life, Social care, Outcomes, Ascot, Proxy

## Abstract

**Background:**

Social care-related quality of life is a key outcome indicator used in the evaluation of social care interventions and policy. It is not, however, always possible to collect quality of life data by self-report even with adaptations for people with cognitive or communication impairments.

A new proxy-report version of the Adult Social Care Outcomes Toolkit (ASCOT) measure of social care-related quality of life was developed to address the issues of wider inclusion of people with cognitive or communication difficulties who may otherwise be systematically excluded. The development of the proxy-report ASCOT questionnaire was informed by literature review and earlier work that identified the key issues and challenges associated with proxy-reported outcomes.

**Methods:**

To evaluate the acceptability and content validity of the ASCOT-Proxy, qualitative cognitive interviews were conducted with unpaid carers or care workers of people with cognitive or communication impairments. The proxy respondents were invited to ‘think aloud’ while completing the questionnaire. Follow-up probes were asked to elicit further detail of the respondent’s comprehension of the format, layout and content of each item and also how they weighed up the options to formulate a response.

**Results:**

A total of 25 unpaid carers and care workers participated in three iterative rounds of cognitive interviews. The findings indicate that the items were well-understood and the concepts were consistent with the item definitions for the standard self-completion version of ASCOT with minor modifications to the draft ASCOT-Proxy. The ASCOT-Proxy allows respondents to rate the proxy-proxy and proxy-patient perspectives, which improved the acceptability of proxy report.

**Conclusions:**

A new proxy-report version of ASCOT was developed with evidence of its qualitative content validity and acceptability. The ASCOT-Proxy is ready for empirical testing of its suitability for data collection as a self-completion and/or interview questionnaire, and also evaluation of its psychometric properties.

## Background

In the UK, social care refers to long-term care services that aim to maintain the quality of life of adults with long-term health conditions or ageing-related support needs (for example, home care, day care or residential care). The increased demand for social care due to ageing populations in Europe combined, in some countries, with the trend to reduce public spending has contributed to an interest in the evaluation of the effectiveness and cost-effectiveness of social care based on the outcomes of people who use services [1]. The shift towards outcomes-based social care policy and administration in the UK has also been shaped by narratives of personalisation, increased choice and control for service users, and wider accountability and transparency in the use of public funds [2, 3].

To evaluate the effectiveness of social care based on personal outcomes rather than outputs, it is important to define the objectives of social care and to develop an appropriate instrument that reflects these objectives. Social care may be broadly conceptualised as services that compensate for the effect of impairments on overall wellbeing or quality of life [4–7]. Therefore, the evaluation of social care support requires the consideration of a broad range of quality of life attributes beyond health that are sensitive to the person-centred, compensatory activity of social care. It has been recognised that condition-specific instruments may not be suitable to assess the broader impact of social care support that is accessed by adults with a wide range of needs and also that generic health-related quality of life measures may not be sensitive enough to capture the outcomes of social care [8].

The construct of social care-related quality of life (SCRQoL) has been proposed as the basis for developing instruments to measure social care outcomes [7]. SCRQoL reflects aspects of quality of life that are important to people who use social care services and may also be conceptualised as the target of the compensatory activity of social care support [9, 10]. The Adult Social Care Outcomes Toolkit (ASCOT) instrument is a preference-weighted measure based on the construct of social care-related quality of life [10–12]. The psychometric properties of the ASCOT self-completion questionnaire (ASCOT-SCT4) have been established in samples of English, Dutch and Australian older adults [12–14]. The instrument has been recognised as a suitable outcome measure for the evaluation of adult social care services [13, 15] and has been used in local and national social care data collections and evaluation studies in England to inform policy strategy, commissioning and care practice [2, 16–18].

If individual quality of life is used evaluate the effectiveness of interventions and policy of social care in this way, a key challenge is how to collect quality of life data from people with cognitive or communication difficulties, who are unable to answer on their own behalf even with support, alternative formats or communication aids, so to avoid the issues of sampling bias and systematic exclusion from ‘having a voice’ [19–21]. In the evaluation of health care interventions using patient-reported outcome or experience measures, a widely-used method is the collection of data from someone who answers on behalf of the individual whose quality of life is to be assessed (by ‘proxy’). Despite its widespread use, it has been argued that data collection by proxy-report should only be used as a last resort when other methods are not possible because it takes away the individual’s opportunity to express their views [22, 23]. While the standard in quality of life measurement is self-report whenever possible, it is recognised that proxy-report is preferable to the systematic exclusion of individuals who are unable to self-report based primarily on the principles of equity and inclusion, as well as the potential methodological issues associated with missing data and bias [19, 20].

The aim of the study was to develop a new social care-related quality of life measure based on the ASCOT-SCT4 that could be used in circumstances where the individual is unable to self-report and other methods of eliciting outcomes information are not feasible. This article outlines the second phase of work that sought to apply the findings identified in the first phase of the project [24] to develop the content and format of the new instrument and to evaluate its content validity and acceptability.

## Methods

This qualitative study aimed to develop a proxy-report version of the self-completion ASCOT-SCT4 with adequate content validity and acceptability to proxy respondents [10]. Content validity is a measurement property that assesses whether questionnaire items reflect the perspective of the population of interest (in this case, proxy respondents for adults who use social care services) and also whether the questionnaire format, wording and instructions are relevant, understandable and acceptable [25]. The study involved an initial phase to develop items and the questionnaire layout based on an earlier phase of research [21, 24] followed by cognitive interviews to refine the questionnaire and establish its content validity [25–28].

### Questionnaire development

While recognising that proxy-report is not equivalent to self-report, the questionnaire development sought to draw upon the same principles and concepts as the ASCOT-SCT4 [10, 12]. The eight attributes captured by the ASCOT-SCT4 are: *Control over daily life; Occupation (‘doing things I value and enjoy’); Social participation and involvement; Personal safety; Personal cleanliness and comfort; Food and drink; Accommodation cleanliness and comfort;* and *Dignity*. The instrument has four response options for each item to distinguish between the ideal state, no needs, some needs and high-level needs in relation to each quality of life attribute [10] (see Table 1).

**Table 1:** ASCOT response levels (adapted from Netten et al. [10])

Response level	Description
Ideal state	The preferred situation, in which the individual’s needs and preferences are met to the desired level
No needs	Where the individual’s needs are met but not to the desired level
Some needs	Where there are needs, but there is no immediate or longer-term health implications
High-level needs	Where there are needs and these have an immediate or longer-term health implication

The key challenges of developing a proxy-report instrument were explored in an earlier phase of research, which involved a rapid literature review, focus groups and in-depth one-to-one interviews [21, 24]. In the English Adult Social Care Survey, the majority of proxy respondents are family or friends who live with the respondent or elsewhere and proxy-report by care staff is relatively infrequent (5.9% of proxy respondents in the 2014/15 ASCS) [29]. It should be recognised that some people may have no or limited contact with family or friends, so care staff may be called upon to act as proxy respondents, especially if they have frequent contact with individuals and in-depth knowledge of the person’s needs and preferences through their practice of care (for example, with support staff who work intensively one-to-one or with small groups of adults with learning disabilities). While some studies have found that proxy-reported quality of life varies by ‘type’ of proxy, for example, health or social care professionals compared to family [20, 30–33], other studies have not found significant differences by type of proxy [34–36]. There is also evidence that this may be accounted for by differences in proximity, intimacy and frequency of contact between the proxy and the individual rather than the ‘type’ of proxy per se [37, 38]. Therefore, in this study and also the earlier phase of research, we considered both care support staff and also family or friends as potential proxy respondents.

The key challenges in proxy response identified in this earlier phase of research were: (1) care workers’ resistance to the idea of acting as a proxy respondent; (2) the perceived difficulty of judging care recipients’ internal subjective state (i.e. how they ‘think’ and ‘feel’); and (3) proxy respondents’ wanted to express that their own response (as a proxy) differed from how they thought the care recipient would respond based on the proxy respondents’ judgement of the care recipients’ perspective [24]. Proxy respondents also commented that they found it difficult to differentiate between the top response options of the ‘ideal state’ and ‘no needs’ [24], whereas earlier work on self-report had found that the inclusion of the ‘ideal state’ supported comprehension and judgement of response [10] (see Table 1). Since the ‘ideal state’ represents that an individual’s needs and preferences are met to his/her desired level [10], it was hypothesised that this was a proxy-specific issue related to the proxy perspective adopted in formulating a response.

In response to the issues summarised above, the authors with advice from the ASCOT development team at the University of Kent (www.pssru.ac.uk/ascot), explored four significant variations in the format and content of the draft proxy version of the questionnaire. First, the questionnaire was formatted to allow the collection of proxy-ratings of quality of life for each attribute from the two proxy perspectives identified by Pickard and Knight [39]. The proxy-proxy perspective represents the proxy’s view based on their own preferences and values, while the proxy-patient perspective asks the proxy to answer based on the proxy’s best attempt at a reconstruction of the individual’s internal mental state based on their knowledge of the individual and their preferences. It was hypothesised that allowing proxy respondents to express both proxy perspectives may improve the acceptability of a proxy-report instrument, especially for paid carers. Furthermore, the specification of the proxy perspective within the questionnaire was anticipated to reduce the potential for bias that may arise if different types of proxy respondents (i.e. paid or unpaid carers), without clear instructions as to which proxy response to use, systematically refer to different proxy perspectives in order to formulate their response to the questionnaire.

The second modification was to include a comments box after each question. In the first phase of work, care staff reported that they would feel more comfortable with acting as a proxy if they had the opportunity to add further detail or explanation of their responses, especially if quality of life had been rated as some or high-level needs [24]. The inclusion of the comments boxes aimed to improve the acceptability of the questionnaire, especially for care workers.

Third, during the development of the original ASCOT-SCT4, it was found that some respondents understood the *Dignity* item to refer to the impact of having help on their self-esteem and self-perception, rather than the effect of the way they are supported or treated by care staff [10]. Based on this evidence, an additional item was developed for the ASCOT-SCT4 to allow respondents to express any difficulties they have with coming to terms with needing help [10]. This item is not considered in scoring the ASCOT-SCT4. In the development of the proxy-report questionnaire, by contrast, it was initially decided to include only one *Dignity* item because of the additional complexity of asking respondents to adopt two different proxy perspectives.

Finally, based on the proxy respondent’s report that they found it difficult to judge how the individual thinks or feels [24], the wording of some items was modified in the draft questionnaire to be more ‘objective’ (version 1.0): for example, *Occupation* was revised to refer to what the person ‘wants to do’ rather than ‘values and enjoys’, *Social participation* referred to any type of social activity rather than specifically to social interaction with ‘people s/he likes’ and the items for *Personal safety* and *Personal comfort and cleanliness* were modified to refer to ‘being’ rather than ‘feeling’ safe or clean and comfortable. In the first phase of interviews with the version 1.0 questionnaire, we sought to explore whether the use of more ‘objective’ criteria would improve the acceptability of the proxy-report questionnaire. The research team were, however, also aware that these modifications were in tension with the underlying conceptual basis of the ASCOT measure to capture social care-related quality of life with reference to the individual’s preferences, values and attitudes.

**Fig. 1:**
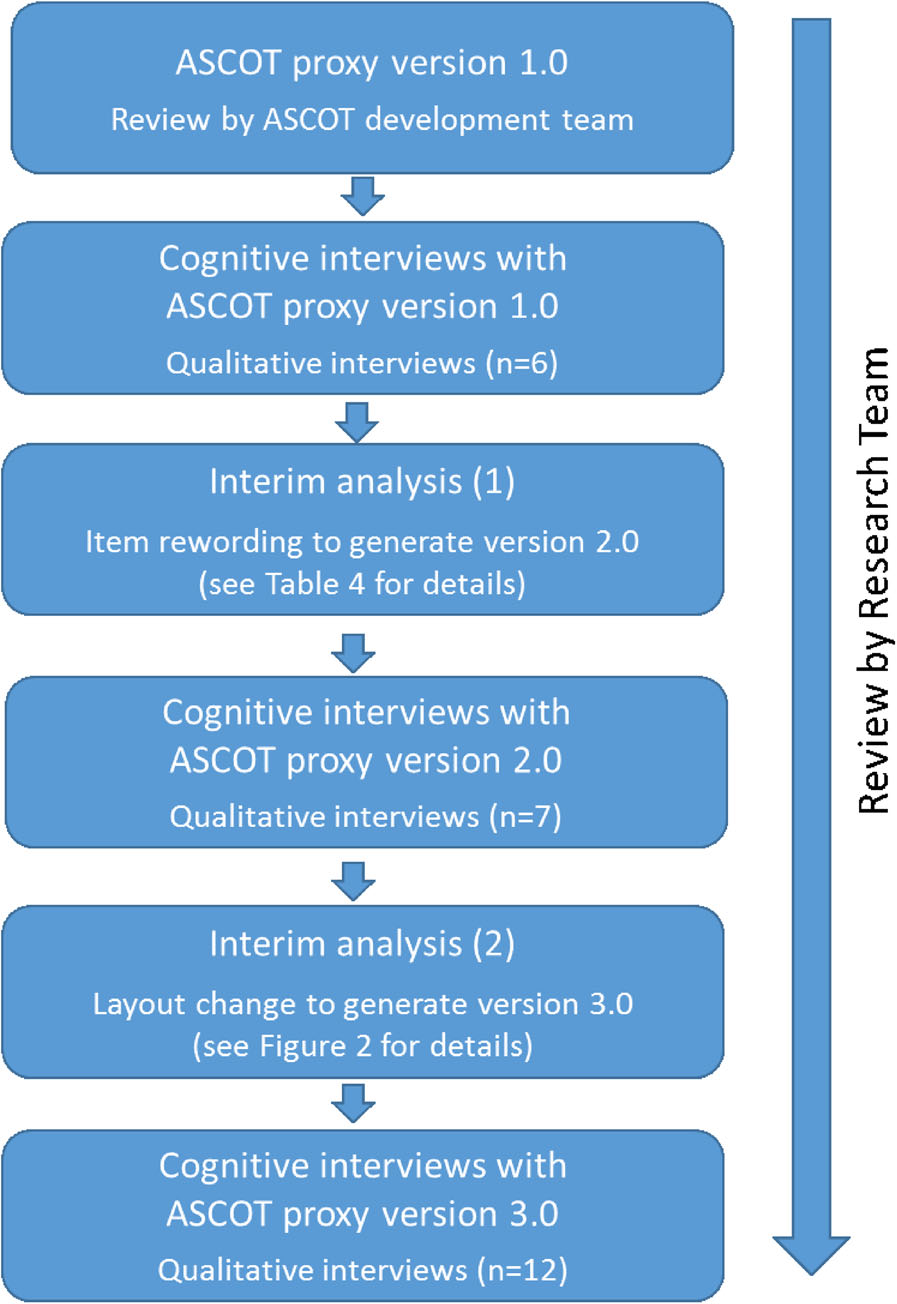
Flow diagram of the process of cognitive testing

A summary of the process of the initial development and refinement of the questionnaire is shown in Fig. 1.

### Recruitment of participants

Participants were recruited purposively via three social care provider organisations across three local authorities in South East England. The study inclusion criteria were adult care workers or unpaid family carers (aged 18 years or over) in regular contact with an adult who uses social care services and who would be unable to answer the ASCOT-SCT4 on his/her own behalf due to cognitive and/or communication impairments. Potential participants were sent an invitation letter and information sheet by the care provider, which explained the purpose of the research. Those who were interested in participating were invited to contact the research team to arrange an interview and to answer any questions about the purpose or nature of the research. A total of 25 proxy respondents (13 care workers, 12 family carers) were recruited and completed a cognitive interview.

The study was approved by the national social care research ethics committee in England (reference: 13/IEC08/0020). Written informed consent was obtained from all participants prior to interview.

### Cognitive interviews

All interviews were conducted between June 2015 and May 2016 by three trained qualitative interviewers (SR, GC, JC), two of whom had prior experience of cognitive interviews in the development of self-report questionnaires (SR, JC). The interviews lasted between 40 and 75 min and took place at a location convenient for the respondent – usually, in a private office or meeting room in the workplace or at the respondent’s home. The proxy respondents were asked to complete the ASCOT questionnaire using a think-aloud method of cognitive interviewing with concurrent follow-up probes [26]. Think-aloud requires the respondent to speak aloud their thoughts as they read the instructions and complete each item of the questionnaire. Following this, the interviewers used verbal probing to further explore the comprehension, clarity and relevance of each item and its response options (for example, ‘why did you choose this response rather than the level above/below?’) [26] (see Table 2 for further examples). The probes were used flexibly by the interviewers to explore the respondents’ understanding and ability to respond to the questions, as well as to identify any potential issues with the acceptability of item wording, format or layout. For some items, alternative versions were presented to assess the proxy respondents’ preferences and to evaluate whether the different content or format would affect the respondent’s comprehension, judgement or response. After the cognitive interview, the respondents were asked to reflect on their overall experience of completing the questionnaire with a focus on clarity and the acceptability of answering the questionnaire as a proxy respondent (see Table 3).

**Table 2:** Examples of cognitive interview probes

• Which answer would you choose? Why?	
• Was it easy or difficult to answer this question?	
• I noticed you were spending some time with that question – can you tell me what you were thinking about?	
• You chose X. Why? How does X differ from Y or Z?	
• I noticed you were looking here, what were you thinking?	
• What does ‘[phrase or word from questionnaire]’ mean to you? Would you be able to explain that in your own words?	
• You answered differently for how you think the person you know would answer, why is that?	
• Could this question be made clearer? If so, in what way?

**Table 3:** Interview guide

• What did you think of the questionnaire?	
• Is there anything you’d like to change? If yes, what would that be?	
• How difficult or easy were the questions to answer? Were any questions particularly difficult/easy?	
• How did you feel about answering this questionnaire?	
• How did you feel about the information you were given at the beginning of the questionnaire?	
o Was this clear?	
o Did it help you to answer the questions?	
o Did you feel worried at all about what would happen to the information collected?	

### Qualitative analysis

The interviews were audio-recorded and transcribed for analysis. The written interview transcripts were analysed using NVivo version 10 software [40]. While it is possible to informally analyse data from cognitive interviews to identify and resolve issues with item wording, layout or format, the use of systematic thematic analysis may provide a more rigorous way of analysing think-aloud cognitive interviews ([26] p.157). The researchers agreed on an initial analytical coding structure based on the framework of cognitive interviews as a technique to explore the understanding and acceptability of questionnaire item wording, format and layout (see Table 4) ([26] pp.164–7). The data was coded by two researchers (SR, GC). The coding structure was iteratively refined based on themes and subthemes that emerged from the data, which allowed the identification of key issues and insights.

**Table 4:** Coding tree

• Introduction	
o Questionnaire format, header and title	
o Who should complete questionnaire?	
o Do you think you should complete the questionnaire?	
• For each ASCOT attribute (Food and drink, Accommodation etc.)	
o Ability to understand the item layout and format	
o Definition of attribute (i.e. what is the question asking?)	
o Comprehension of particular words or concepts	
o Ability to formulate a response and report an answer	
▪ Understanding of the response options	
▪ Difference between proxy perspectives (proxy-proxy, proxy-patient)	
▪ Comprehension of adequate vs. enough (if applicable)	

### Iterative questionnaire refinement

The questionnaire was refined through an iterative process of cognitive interviewing ([26] p.146). Each round of interviews had a minimum of six and a maximum of twelve participants, which is within the guidelines suggested by Willis [26] (p.138). The researchers met during the fieldwork to review the data, to discuss any emerging themes, and to agree on any modifications to the questionnaire.

After six interviews with questionnaire version 1.0, the data were analysed to identify any issues with comprehension, response or acceptability that would justify modification of the questionnaire at that point. The edits were applied to generate version 2.0 of the questionnaire, which was tested in the next round of interviews (*n* = 7). The second interim analysis considered whether any further changes were required based on the interview data. The questionnaire with these changes is referred to as version 3.0, which was then tested in the final set of interviews (*n* = 12). During this final set of interviews, the research team agreed that we had reached saturation (i.e. no new concepts or issues emerged). At this stage, the third and final analysis was conducted of this final set of interviews, both separately and also alongside the two earlier phases.

A summary of the iterative development process is shown in Fig. 1.

## Results

### Sample characteristics

The demographic characteristics of the sample are shown in Table 5. Of the 25 respondents, 60% were female. The sample comprised care staff (*n* = 13) or family carers (*n* = 12). Half of the family carers responded on behalf of their spouse or partner (*n* = 6). The other carers responded on behalf of other family members (their adult children, parents or siblings). The majority of proxy respondents reported that the care recipient’s primary support reason was an intellectual disability (*n* = 9) or autism (*n* = 7). The remaining interviews were conducted with proxies who spoke on behalf of someone with dementia or another long-term condition that affected cognitive ability, comprehension or ability to respond.

**Table 5:** Characteristics of the cognitive interview participants (*n* = 25)

	Frequency (%)
*Gender*	
Male	10 (40%)
Female	15 (60%)
*Type of proxy respondent*	
Formal (care worker)	13 (52%)
Informal (family)	12 (48%)
*Primary support reason of cared-for person*	
Intellectual disability	9 (36%)
Autism	7 (28%)
Dementia	6 (24%)
Other (stroke, pain/confusion)	3 (12%)
*Living situation of cared-for person*	
Community (owner-occupied/rented accommodation)	7 (28%)
Supported living	11 (44%)
Residential or nursing care home	7 (28%)

### Cognitive interviews

With the iterative modifications outlined in detail below, the questionnaire items were found to be understandable and acceptable to care staff and family carers asked to act as proxy respondents. A summary of the findings in relation to the understanding of concepts and acceptability of each item to proxy respondents is presented in Table 6.

**Table 6:** Ease of understanding and acceptability of the ASCOT-Proxy items

Attribute	Correct interpretation of item	Acceptable to proxy respondent	Example quote(s) to illustrate understanding of concepts
Food and drink	25/25	25/25	“I was thinking about the individual that I was thinking of, and thinking about what she eats and what she likes.” [GC_FC_01] “I feel in my opinion she got all the food and drink that she wanted and I feel she would answer exactly the same.... She couldn’t communicate unless you really did extra work, if you put that extra work in. So around choice when I started working with her it was going out into the community and going round the shop and asking her to point to the things that she would like on her shopping list and then actually forming a list that was hers through all her choices. And then it was about looking at health and what would be good for her health and then it was negotiating – maybe should some of the junk food reduce? So it was over quite a protracted time to get to the stage of finding out exactly what she wanted. So by the end of that process I would say she got anything she wanted when she wanted it… I would say if someone’s communication is really limited to just making verbal sounds actually knowing when they would want a drink or something to eat it’s going to be that close--, very close relationship where the carer actually knows by a certain sound whether they’re actually requesting something.” [JC_FC_03]
Accommodation	22/25^a^	25/25	“She has her own room, use of a bathroom, she goes to the kitchen, she likes to sit in the conservatory, and listen to her music… she’s got freedom to do what she wants to in comfort, and yeah, it’s warm and clean.” [GC_FC_05] “They hoover every day. The kitchen is spotless. The bathroom is spotless. The utility room is spotless. It has a toilet that’s all spotless. Everything--, the dining room is spotless. The lounge sometimes because they--, because of the way they are. I mean [Name]‘s fairly tidy but there’s two other residents there and one of them is not particularly tidy. So when you go there and it looks it’s a bit of a mess like, you know, why is it like this? What can the carers do? They can’t be behind that person with a dustpan or hoover all the time, can they? You know, it’s just how things are. So sometimes, no. But by the end of the day -- if you go there in the evening or if I go out late with [Name] and come back late [the other residents] are in bed, that lounge is then spotless.” [SR_IC_02]
**Personal comfort and cleanliness**	25/25	25/25	“A: It’s not what I feel, it’s how they feel, yeah. And I’m hoping that they feel that they are clean and presentable when they-- Q: And do you get that impression? A: Yeah, because they’re happy. One of the ladies when she’s got perfume on she’ll giggle and give you that sort of, when you say, “You look lovely,” and you’ve brushed her hair she’ll give you a little giggle and chuckle -- and smile and so you know that she’s quite happy with the way she’s dressed.” [GC_FC_05] “I’d say less than adequately clean and presentable for us because he needs continual prompts for personal care and clothing. He’s very, very funny with clothing. You’ll never get him in pairs of trousers or jeans or anything like that, tracksuit bottoms and t-shirts all the time. He won’t bath. He won’t shower. He just has a wash, and we don’t know if he’s doing it properly.” [SR_IC_03]
Social participation	25/25	25/25	“Because he’s quite mute, he’s a man of few words, so he doesn’t socialise. He does discos, but he doesn’t have any friends as such. He has his friends and he has his family that contact him. He goes home on a home visit quite a bit but apart from that, that’s about it because he’s not very--, I think he finds it hard to maybe make friends. He’s got a few, obviously on site, where he’s been here years.” [GC_FC_02] “…because they are in their own little worlds, because they will say hello to people if they--, but they very rarely mix with people, they may go over there to a disco but they’re still in their own little group, so it’s very difficult with people like that because of their autism and their behaviours they feel safe in their own little thing, even though we include them in everything, so they can go out, the cinema, swimming, in the public, go bike riding, they do the clubs here and everything else. But I wouldn’t even say they socialise with each other… I think the one thing we lack is actually still building on relationships, and friendships and things like that, sometimes that’s not just down to us, sometimes that might be due to the care manager cutting hours and they haven’t got that, you know, they’re not paying for social activities. They’re actually paying for care, you know.” [JC_FC_01] “Not many people come to see her… visitors have stopped. She gets very few visitors apart from family. But her church is at the end of her road and that is very near. She can go there, which she does and takes a friend every Sunday.” [SR_IC_11]
**Occupation**	25/25	25/25	“I’d say ‘values’ maybe something that is dear to them, maybe it’s a pastime that they enjoy, like something personal that is enjoyable with a friend or family or even by themselves. Enjoys and they do it whole heartedly something that means a lot to them.” [GC_FC_07] “She’ll sit and watch DVDs, listen to music and dance, sit in the lounge… there is very clear stuff that she really enjoys and that she will go, she’ll be early for that activity.” [GC_FC_01] “Well she plays computer games in the morning. And it’s a fairly set routine, we have breakfast, she plays on the computer until she gets fed up… until it’s Bargain Hunt or some equally bloody stupid thing on the television [laughs]. And then it’s lunch time, then it’s Neighbours. Then it’s possibly cross stitching until it’s Homes in the Country or something, oh, just some rubbish [laughs]. And I disappear. I can go without the television.” [SR_IC_09]
Control over daily life	25/25	22/25^†^	“He chooses for example what he wears, what time he gets up, what he gets to eat, activities we will try to do two activities a day with him, obviously he will get that choice what he wants to do, I mean as if he wants to ring mum, dad, or whatever, you know, he’s got control of that, so yeah he does have control over a lot of things.” [GC_FC_06] “It’s like that’s [i.e. not always having full control or choice over daily life] the norm for them, which is not always the best, but at least they’re comfortable and their interests are being addressed.” [GC_FC_07] “They’re never going to have total control over their life, if I’m thinking of [name], he’s never going to have total control over his life, ‘cause his disability does not allow that, so that shouldn’t be as much control over his--, as he or she wants. It’s the ability, as much as possible, has control over his or her daily life.” [JC_FC_01] “I say she’s got as much control as she wants. Well she has, because she does what she wants… She’s very happy to let me do all those sort of things [organise financial affairs] because she can’t understand them… It’s sometimes difficult to decide how much I should do and how much we let her try and do. Obviously she wants to do as much as she can, but sometimes it doesn’t happen. But I think she has as much control as she wants to have. I don’t think she wants a lot of control, really.” [SR_IC_11]
**Personal safety**	25/25	25/25	“I was thinking about how she is and how she will hold onto you and hold onto your hand if she’s out in the community for reassurance and to feel safe and because she’s supported by people who know her.” [GC_FC_01] “Safe in the house, they can lock the door and it’s, that’s it, you know, because there are a few not nice people around this particular area and it’s, yeah. We’ve never had any problem with anyone and they feel, they know they shut that door and, yeah, that’s it. The outside world’s out there, I’m in here. It’s nice and cosy, yeah. I think so, definitely… they feel safe in themselves”. [SR_FC_03] “The only time that she doesn’t feel safe is when they’re having to turn her because they’re repositioning her, or washing her. Even when she had a lot more cognitive ability she--, even a simple turn in the bed, −- she would imagine that she’s falling, and she’d say, “Oh be careful, I don’t want to fall,” not realising that she’s in the bed and that the bed’s got supports, that she can’t fall… I noticed last week and the week before she wasn’t really saying that, so I guess she must be feeling quite safe now, you know, so in my opinion and her opinion and I would say--, and we certainly don’t think that she’s not safe, you know?” [SR_IC_04]
**Dignity**	22/25^b^	18/25^††^	“Being respected, making sure you’re not--, you’re fully clothed, stuff like that. Thinks and feels better--, yeah I think being smart, being respected, yeah.” [GC_FC_02] “The fact that he has the care helps him… he would feel vulnerable, yes. But he knows that ‘so and so is supporting me’, so he’s very comfortable on that.” [JC_FC_02] “If he wakes up in a wet bed he gets embarrassed. If the staff are very used to him, very experienced staff, then they could get him out of that quite easily. If you’ve got staff that aren’t and, you know, don’t really know how to approach him, then it becomes a big issue.” [SR_IC_01] “And they [care staff] all treat him in a way that’s going to make him feel better, the way they talk to him.” [SR_IC_02]

Bold: Item modified after round 1 of cognitive interviews (see Table 7 for further details)

^a^Three proxy respondent included aspects of personal appearance (e.g. washing self) in their evaluation and response to the question; however, once they realised the next question asked specifically about *Personal comfort and cleanliness*, the respondents reviewed their response to the *Accommodation comfort and cleanliness* item. Based on this, no revisions were made to this item

^b^Two family carers included the effect of unpaid care they provided in their evaluation and response to the question**.** The question was revised to make it clearer that it refers only to the effect on paid care on how the person thinks and feels about him/herself (see Table 7 for further details)

^†^One proxy respondent (care worker) felt uncomfortable with ‘control’ because it had negative connotations of control over someone. Two proxy respondents (one care worker and one family carer) noted that, as a result of the person’s condition (autism), the person wanted more control than would be realistically possible. As such, they would not be able to answer ‘as much control as s/he wants’

^††^One care worker felt that the question may result in care workers answer as they thought the service would want them to, rather than as an honest reflection of their own opinion (proxy-proxy perspective) or their view of the person’s opinion (proxy-patient perspective). One care worker found it difficult to rate the proxy-patient perspective; however, the respondent was not able to articulate why. Three proxy respondents (one care worker, two family carers) said that they found it difficult to rate *Dignity* from the proxy-patient perspective because of condition-specific considerations related to learning disability and/or autism that meant the person lacked self-awareness and, therefore, did not have the ability to think or feel about themselves. Two respondents found it difficult to answer the question because they person they were representing did not currently receive help or support from paid care staff in any context

### Interim analysis and review (1)

Based on analysis of the first round of interviews, the modifications outlined in Table 7 were applied to the draft questionnaire. For *Occupation* and *Social participation*, it was found that the wording of the original ASCOT-SCT4 item or response options were preferred to the version 1.0 proxy questionnaire because they were perceived to be more person-centred (*“looks at the individual”*). One formal carer, for example, compared the two versions of the *Occupation* item, ‘what s/he values and enjoys’ (original ASCOT-SCT4) to ‘what s/he wants to do’ (version 1.0), as follows:

*“I think that’s worded better… because it’s talking about her values and her enjoyment… When we’re looking at the people we support we’re looking at their values and what they like to do, their likes and dislikes - so I just think it’s easier to answer”. *[GC_FC_01]

**Table 7:** Modifications based on respondent feedback (questionnaire version 1.0)

Item/attribute	Rationale for modification
Introduction	No modifications.
Food and drink	No modifications.
Accommodation comfort and cleanliness	Response option (some needs) changed from *‘Not quite clean or comfortable as s/he would like’* to *‘Not quite clean or comfortable enough’* to improve the proxy respondent’s comprehension and ability to distinguish between response options.
Personal cleanliness and comfort	Item wording changed from *‘the person I am representing is…’* to *‘the person I am representing feels…’* for consistency with the ASCOT-SCT4 (standard). ^a^
Social participation and involvement	Response option (ideal state) changed from *‘As much social contact with people as s/he wants’* to *‘As much social contact as s/he wants with people s/he likes’* to improve acceptability and comprehension. Response option (no needs) changed from *‘Enough social contact with people. It’s OK’* to *‘Enough social contact with people’* to improve acceptability of the item. (The respondents felt that the *‘It’s OK’* was not needed and sounded too informal).
Occupation	Item wording changed from *‘Which of the following statements best describes how the person you represent spends his/her time? This includes anything that s/he does in his/her day-to-day life’* to include at the end *‘that s/he values and enjoys’* to reflect the respondents’ preference for the inclusion of these words. ^b^ Response options (all) changed from referring to what the person *‘wants to do’* to what she *‘values and enjoys’.*
Control over daily life	No modifications.
Personal safety	Item wording changed from *‘the person I am representing is…’* to *‘the person I am representing feels…’* for consistency with the ASCOT-SCT4 (standard). ^a^ Response option (no needs) changed from *‘Safe. It’s ok’* to *‘safe’.* (The respondents felt that the *‘It’s OK’* was not needed and sounded too informal). Response option (ideal state, no needs) changed from *‘very safe’* and *‘safe’* to *‘as safe as s/he wants’* and *‘adequately safe but not as safe as s/he would like’* to improve comprehension, the proxy respondent’s understanding and ability to distinguish between response options.
Dignity	Item format changed to bold and italicise ‘paid carers’. Item wording changed from *‘Because of the care s/he receives…’* to *‘Because of the paid care s/he receives…’* to improve comprehension that the question relates only to the effect of paid care.

^a^The *Personal cleanliness and comfort* and *Personal safety* items were amended to reflect the concept of ‘feeling’, rather than ‘being’, clean and presentable or safe. This was based on finding that the respondents in the first round of interviews were intuitively reflecting on how the person they were representing *feels and thinks* (i.e. from a subjective perspective) when answering from the proxy-patient perspective (for example, “He will hold your hand when you’re on the street because he feels safe doing that… he knows he’s safe in the house. He knows that we’ll protect him, under no circumstance will he ever get hurt.” [JC_FC_01]

^b^In the first round of interviews, the proxy respondents were asked to look at both item wordings and to say which they preferred, and also why. Four of the six respondents in the first round of cognitive interviews stated a preference for the inclusion of ‘that s/he values and enjoys’: for example, “that’s much better. That’s a much better question… I think that’s worded better… because it’s talking about her values and her enjoyment. It’s not what you want to do, it’s what--, it’s another way of wording what you want to do. But actually when we’re looking at the people we support we’re looking at their values and what they like to do and their likes and dislikes and everything so I just think it’s easier to answer.” [GC_FC_01]. One respondent did not specify a preference. The sixth respondent expressed a qualified preference for the inclusion of ‘that s/he values and enjoys’: “I like the word enjoy, because when people have got that lesser communication and they can’t do it, we put activities on what they enjoy. We can evidence that through behaviours and smiley faces and things like that, so I do like the word enjoys… but I’d just put and enjoys (not values).” [JC_FC_01]

Because of this, the items of *Occupation, Social participation, Personal safety* and *Personal comfort and cleanliness* were modified to correspond to the original ASCOT-SCT4 item wording to improve acceptability and comprehension (see Table 7).

Based on the earlier research that explored care staff and carers’ views on the ASCOT-SCT4 questionnaire, the version 1.0 proxy questionnaire sought to avoid the use of ‘enough’ because of potential issues with comprehension or acceptability [24]. The first round of interviews identified that respondents found the alternative wording used in version 1.0 (‘It’s ok’) to be too informal and imprecise:

*“I just don’t like how that looks, just remove the ‘it’s ok’… it’s too vague”. *[SR_FC_02]

The first round of interviews explored whether the adaptation of the *Personal safety* and *Personal comfort and cleanliness* items to be more ‘objective’ (i.e. *being* rather than *feeling* safe) would improve acceptability of the measure to proxy respondents [24]. Interestingly, despite the finding of the earlier phase of work that some respondents were uncomfortable with rating the cared-for person’s internal subjective state, the respondents spontaneously answered the questions by attempting to construct the cared-for person’s subjective perspective based on their observations and knowledge of the individual. This was prompted by the questionnaire format of asking respondents to rate the two different proxy perspectives (proxy-proxy and proxy-patient).

*“It took me a little bit longer to answer this one I thought, I don’t know whether you thought exactly the same but because obviously I’m trying to think how this individual is thinking, and also I'm thinking about what she does, and is that because she doesn’t feel 100 percent safe, and what does safe mean to her as well”.* [GC_FC_01]

Based on this finding, the *Personal safety* and *Personal comfort and cleanliness* were adapted to correspond to the original ASCOT-SCT4, which refers to *feelin*g rather than *being* safe or clean and presentable. In subsequent rounds of cognitive interviews, the respondents were able to understand and respond to these two modified items with no evidence of any issues with acceptability.

### Interim analysis and review (2)

Based on advice from the ASCOT development team, the researchers asked respondents in the second round of cognitive interviews to compare two versions of the questionnaire (see Table 8). Cognitive interviews with a carer version of the ASCOT, the ASCOT-Carer, had identified that some respondents were not able to understand the word ‘adequate’ used in some response options [41]. The aim of comparing the two versions in this study, which used either ‘adequate’ or ‘enough’ in the response options for six of the eight items (see Table 8), was to further test the acceptability and comprehension of ‘adequate’ and also to determine whether the alternative item wording (‘enough’) would affect comprehension, judgement or response in the context of a proxy-report questionnaire. For each of the six items, the respondents were asked to respond to the version using ‘adequate’ before being asked to review the version using ‘enough’. The interviewer probed to explore whether the respondent preferred one version to the other, and also whether the different wording affected the respondent’s comprehension, judgement or response.

**Table 8:** Questionnaire version 2.0 modifications to response options

Item/domain	Response options	ASCOT-SCT4 version (‘adequate’)	Alternative version (‘enough’)
Food and drink	No needs Some needs High-level needs	Gets *adequate* food and drink at OK times. Doesn’t always get *adequate* or timely food and drink. Doesn’t always get *adequate* or timely food and drink, and there is a risk to his/her health.	Gets *enough* food and drink at OK times. Doesn’t always get *enough* or timely food and drink. Doesn’t always get *enough* or timely food and drink, and there is a risk to his/her health.
Accommodation comfort and cleanliness	No needs	Is *adequately* clean and comfortable.	Is clean and comfortable *enough.*
Personal cleanliness and comfort	No needs Some needs	*Adequately* clean and presentable. Less than *adequately* clean or presentable.	Clean and presentable *enough.* Not clean or presentable *enough.*
Social participation and involvement	No needs	*Adequate* social contact with people.	*Enough* social contact with people.
Control over daily life	No needs	*Adequate* control over his/her daily life.	*Enough* control over his/her daily life.
Personal safety	No needs Some needs	*Adequately* safe, but not as safe as s/he would like. Less than *adequately* safe.	Safe, (but not as safe as s/he would like). Not safe *enough*

There was no clear preference between the two versions. Two respondents showed a consistent preference for ‘adequate’ [GC_FC_05; SR_FC_03], while three respondents preferred ‘enough’ [GC_FC_04; GC_FC_06; SR_IC_01; SR_IC_02] and one respondent indicated a preference for ‘adequate’ or ‘enough’ that varied by item [SR_IC_03]. The findings indicate that ‘adequate’ was understood by some respondents to be an objective baseline related to social services’ or other external agencies’ standards, while ‘enough’ implied a subjective standard.

*“If you’re social services… they’ve got very different opinions of what’s adequate to bring a child up in a house. Most of us would say definitely not, but because there’s levels, there’s only concerns on certain levels, isn’t there? So adequate makes me feel that that’s what it’s looking at, whether it’s adequate. Whereas this is more about--, if you’re asking my opinion then it’s whether it’s enough for me”.* [SR_IC_01]

While the intention of the ASCOT tools is to ask respondents to rate quality of life from a subjective perspective rather than an objective standard, it was decided to use the term ‘adequate’ rather than ‘enough’ to maintain comparability between the ASCOT-SCT4 and the proxy-report version.

### Proxy perspectives

Pickard and Knight’s (2005) conceptual framework of proxy response was used to develop proxy questionnaire items that asked: (1) respondents to rate their own view (proxy-proxy perspective); and (2) what they thought the care recipient would say (proxy-patient perspective). This dual response approach was found to be acceptable to care worker and family carer proxies. The format allowed the respondents to express differences between their opinion and their understanding of the care recipient’s perspective. These differences were explained by reference to different personal preferences influenced, for example, by social or cultural norms or health condition-related factors:

*“Her generation don’t do all this changing sheets every day malarkey and they don’t believe in so much washing. That’s just alien to them. And she used to say, “That’s fine, it doesn’t need a wash”. But to me it would be like reeking and I’d be like, “No, you must”.* [SR_IC_10]

**Fig. 2:**
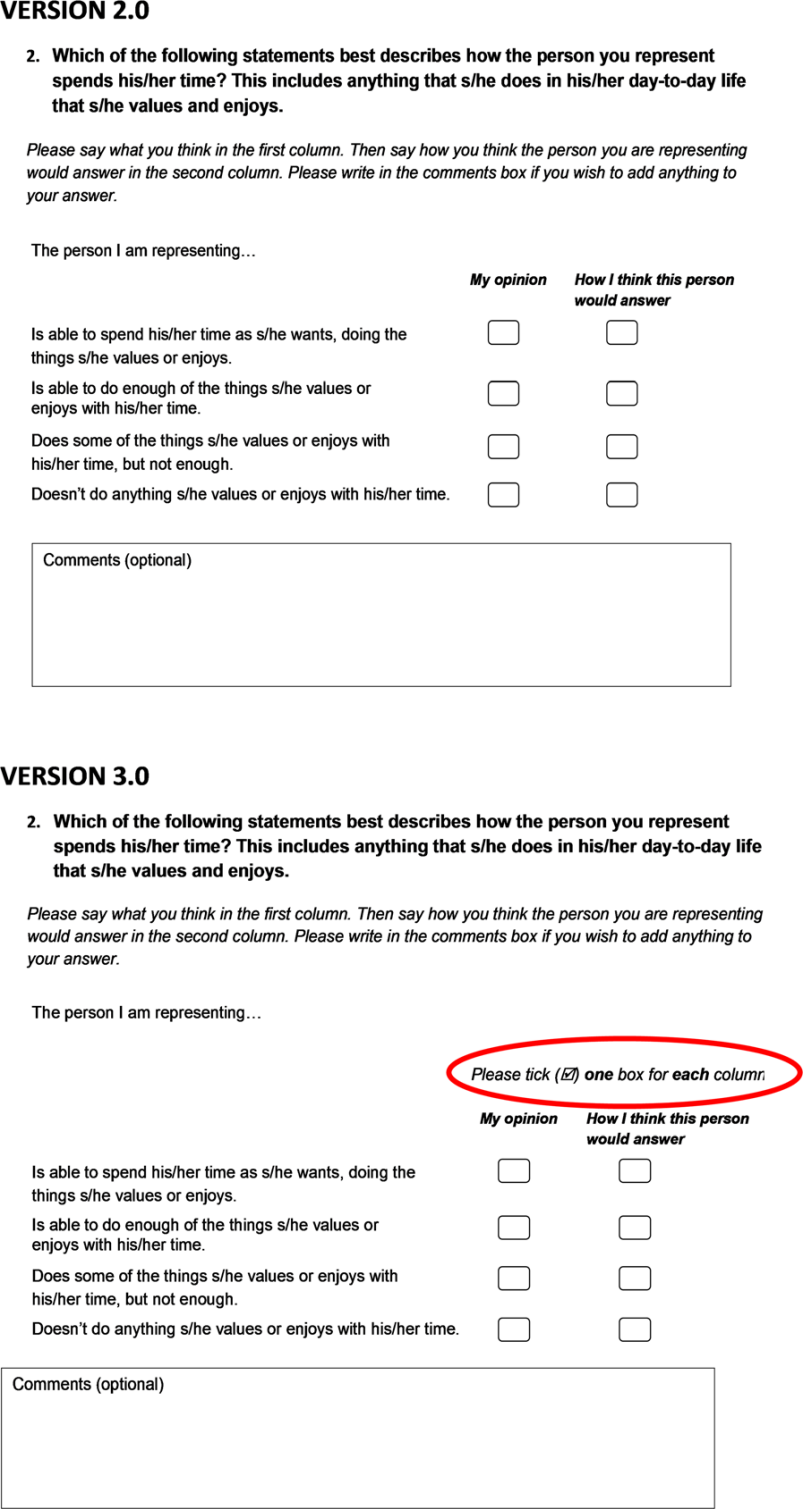
Questionnaire format

While the results suggest that the two proxy perspectives improved the acceptability of the questionnaire, some respondents were confused by the questionnaire layout with two columns that correspond to the two proxy perspectives (see Fig. 2). In the first two rounds of interviews, four of the thirteen proxy respondents answered the first question (*Food and drink*) by ticking multiple boxes in each column or writing yes/no to indicate whether each statement applied or not. They were, however, able to correctly complete the remainder of the questionnaire once they had realised that the format required one tick per column. Based on these findings, the questionnaire for version 3.0 was re-formatted to include ‘please tick one box’ above the response boxes (Fig. 2).

In the third round of interviews (*n* = 12), two family carers were initially confused by the layout. The interviewers had been briefed to allow the respondent to continue the questionnaire without prompts to highlight the questionnaire instructions to tick one box per column. In one of the two cases ([SR_IC_05]), the respondent spontaneously realised that their initial response with multiple ticks had been incorrect and amended their response. There were no issues with the remaining questions. In the other case ([SR_IC_07]), the respondent directly asked the interviewer whether they needed to tick multiple boxes in each column. Once they had seen the instruction, with a prompt from the interviewer, the respondent understood that only one tick per column was required to indicate their rating:

*“Tick, ‘Please tick one box for each,’ oh, ‘Tick one box for each column.’ Oh I see, so [sigh], so it’s just the top one then isn’t it?”* [SR_IC_07]

### Dignity

The ASCOT *Dignity* item aims to capture the positive and negative psychological effects of formal support and care on the service user’s personal sense of significance [10]. In this study, it was found that the *Dignity* item was generally understood to relate to the effect of care workers' working practices and interpersonal interactions on the cared-for person’s sense-of-self. The interviews often focussed on interpersonal interactions associated with personal care:

*“I would say dignity is like--, if someone is having a bath and you’re bathing them, like for instance they get out of the bath you turn away or you give them towels so they can hide themselves so they’ve, you know, privacy”.* [SR_FC_02]

Other respondents, however, reflected on the wider implications of care practice and the quality of interpersonal interactions with care staff on the individual’s self-perception and dignity:

*“I would say the quality of care is what results in dignity and respect… If the person who is going in to support them ultimately has that person’s--, as top most priority their needs, they will always think better of themselves at the end of that”.* [JC_FC_03]

In one interview with a family carer, although the carer initially answered the question in relation to paid care, the boundary between the support given by family and paid carers became blurred:

*“I would probably tick the top one and say he thinks and feels better about himself because we always do, we always praise him up. We always tell him he looks good”.* [SR_IC_03]

To address this issue, the question format was modified to bold and italicize the reference in the question to *paid care*.

As observed in the development of the ASCOT-SCT4 [10], although less frequently, three proxy respondents (care worker (1), family carer (2)) spoke of the effect of needing help on the individual’s sense-of-self.

*“Have I ever met anyone who has been quite positive and upbeat? I don’t think I ever have. I think if I did they most probably wouldn’t want our support … it’s the very act of receiving care that may make them feel undermined”.* [JC_FC_03]

Importantly, however, the ‘think aloud’ indicated that the three proxy respondents made their final judgement and response based on the way in which the care recipient was treated by care workers, rather than the effect of needing help on the individual’s sense-of-self.

## Discussion

The aim of this research was to develop a proxy-report measure of social care-related quality of life and establish its qualitative content validity through cognitive interviews [26]. This qualitative research showed that proxy respondents were generally able to respond to an adapted version of the ASCOT-SCT4 instrument designed to capture social care-related quality of life. The acceptability of the questionnaire was improved by the use of the two proxy perspectives proposed by Pickard and Knight [39] to allow respondents to express their opinion (*proxy-proxy perspective*) and distinguish this from their view of the care recipient’s own perspective (*proxy-patient perspective*). The findings of this study indicate that proxy respondents found it acceptable to adopt both proxy-proxy and proxy-patient perspectives and that they were able to understand and respond to the items based on both perspectives.

While an earlier phase of work found that care workers were hesitant to provide proxy judgements of individual care recipient quality of life especially when questions related to subjective concepts such as *feeling* safe rather than more objective judgements, such as *being* safe [24], this qualitative study indicates a high-level of acceptability of judging subjective states using the two proxy perspective conceptual framework [39]. Furthermore, the first round of interviews indicated that the item modifications that made the items less subjective in description (i.e. *being* rather than *feeling* safe) were less acceptable than items framed around the individual’s subjective perspective when proxy respondents were given a questionnaire based on dual proxy-patient and proxy-proxy perspective report. However, some proxy respondents still had difficulty in projecting themselves into the individual’s internal state when adopting the proxy-patient perspective, especially when the proxy respondent had to rely on ambiguous external cues and behaviours to formulate their response.

While the two proxy perspective methodology has improved the acceptability of proxy report of social care-related quality of life in the eight ASCOT-SCT4 domains [24], there remains the question of which source of information to use if the aggregate data is to be used for policy, administration and planning of social care services. The proxy-report framework proposed by Pickard and Knight [39] was developed in the context of health-related quality of life alongside a differentiation between the intra-rater gap between proxy perspectives and also the inter-rater gap between self-rated and proxy-rated quality of life. Indeed, there is an extensive literature that compares self-report and proxy-report, of which only a minority of studies have found no significant difference between self-report and proxy-report [42–44] or that proxy-report tends to overestimate quality of life compared to self-report [45]. The majority of studies that directly compare self-report and proxy-report have found an underestimation of quality of life by proxy respondents compared to patient self-report (for example, [20, 34, 38, 46–65]. While it is unclear whether these differences represent a systematic bias in proxy-report that may be extrapolated to cases where proxy-report is the only means of estimating an individual’s quality of life [19], it is evident that proxy-reported quality of life cannot simply be used interchangeably as a substitute for self-reported quality of life.

The two proxy perspectives may be conceptualised as two separate sources of information based on the proxy respondent’s mental construct of the care recipient’s likely internal state informed by their knowledge of the individual (*proxy-patient perspective*) or the proxy’s judgement based on their internal standards, preferences and attitudes (*proxy-proxy perspective*) [39]. Based on this view, it has been proposed that in the absence of a clear justification of one over the other, it would be informative to gather ratings based on both perspectives [21, 39]. When collecting data from both proxy perspectives, there remains the judgement as to how to use the two sets of data in analysing, reporting and applying the data in policy-making and service improvement. As such, while the qualitative analysis presented in this paper has shown that proxy respondents are able to rate both proxy perspectives and that this improves the acceptability of the questionnaire, further work is required to establish the psychometric properties of the data collected from both proxy perspectives and to explore how best to handle, analyse and apply the data.

The study has some limitations. First, the first phase of cognitive interviews included only care workers who supported adults with intellectual disabilities in a supported living context. In the subsequent two rounds of interviews, we purposively sampled a mixture of care workers and family carers because of evidence that different types of proxy respondent may respond differently to proxy-report questionnaires [66, 67]. We also sought to include carers or care staff who supported adults with other support needs and who lived in different support contexts to ensure that the questionnaire was feasible and acceptable to a range of proxy respondents. Second, the testing of the final version indicated that not all respondents were immediately able to understand the layout of the questionnaire. While there was no evidence of comprehension issues related to the two proxy perspectives, some respondents assumed that they had to tick all boxes in each column that applied rather than tick one box to indicate which statement best applies. This issue persisted even with a modification to include the instruction to ‘please tick one box for each column’ above the response options (see Fig. 2). Although it was found that respondents were eventually able to work out how to record their response to the questions, a pilot self-completion survey would be required to explore whether proxy respondents would be able to work through this without the presence of an interviewer in the context of a paper-based self-completion survey.

## Conclusions

In conclusion, it was found that the item wording, format and layout of the ASCOT-Proxy questionnaire was understandable and acceptable to care workers and family carers invited to act as proxy respondents. Respondents’ comprehension of items corresponded to the construct definitions captured by the items in the self-report version of ASCOT-SCT4. This was improved by modifications to the ASCOT-Proxy to minimise differences in item wording between the ASCOT-Proxy and ASCOT-SCT4, whilst maintaining the acceptability of the instrument. The next step to evaluate the feasibility and psychometric properties of the new instrument in the context of self-completion surveys is justified.

## Abbreviations

ASCOT: Adult social care outcomes toolkit
ID: Intellectual disability
LA: Local authority
QoL: Quality of life
SCRQoL: Social care-related quality of life
SCT4: Self-completion, four-level questionnaire

## References

[1] Waldhausen A (2014). Care services in crisis? Long-term care in times of European economic and financial crisis.

[2] Department of Health (2011). Transparency in outcomes: a framework for adult social care.

[3] Bovaird T (2012). Attributing outcomes to social policy interventions: ‘gold standard’ or ‘fool's gold’ in public policy and management?. Soc Pol Adm.

[4] Nocon A, Qureshi H (1996). Outcomes of community care for users and carers: a social service perspective.

[5] Qureshi H, Patmore C, Nichols E, Bamford C (1998). Outcomes in community care practise. Overview: outcomes of social care for older people and carers.

[6] Bamford C, Qureshi H, Nicholas E, Vernon A (1999). Outcomes in community care practice: outcomes of social care for disabled people and carers.

[7] Netten A, Beadle-Brown J, Caiels J, Forder J, Malley J, Smith N, Towers A, Trukeschitz B, Welch E, Windle K (2011). Adult social care outcomes toolkit (ASCOT): main guidance v2.1.

[8] Forder J, Caiels J (2011). Measuring the outcomes of long-term care. Soc Sci Med.

[9] Netten A (2011). Overview of outcome measurement for adults using social care services and support.

[10] Netten AP, Burge P, Malley J, Potoglou D, Towers A, Brazier B, Flynn T, Wall B (2012). Outcomes of social care for adults: developing a preferences weighted measure. Health Technol Assess.

[11] Potoglou D, Burge P, Flynn T, Netten A, Malley J, Forder J, Brazier J (2011). Best-worst scaling vs discrete choice experiments: an empirical comparison using social care. Soc Sci Med.

[12] Malley J, Towers A-M, Netten A, Brazier J, Forder J, Flynn T (2012). An assessment of the construct validity of the ASCOT measure of social care-related quality of life with older people. Health Qual Life Outcomes.

[13] Van Leeuwen K, Bosmans J, Jansen A, Hoogendijk E, van Tulder M, van der Horst H, Ostelo R (2015). Comparing measurement properties of the EQ-5D-3L, ICECAP-O, and ASCOT in frail older adults. Value Health.

[14] Kaambwa B, Gill L, McCaffrey N, Lancsar E, Cameron I, Crotty M, Gray L, Ratcliffe J (2015). An empirical comparison of the OPQoL-brief, EQ-5D-3L and ASCOT in a community dwelling population of older people. Health Qual Life Outcomes.

[15] Makai P, Brouwer W, Koopmanschap M, Stolk E, Nieboer AP (2014). Quality of life instruments for economic evaluations in health and social care for older people: a systematic review. Soc Sci Med.

[16] Forder J, Jones K, Glendinning C, Caiels J, Welch E, Baxter K, Davidson J, Windle K, Irvine A, King D, Dolan P (2012). Evaluation of the personal health budget pilot programme.

[17] Johnstone L, Page C (2013). Using Adult social care outcomes toolkit (ASCOT) in the assessment and review process. Research Policy Planning.

[18] Department of Health (2014). The Adult social care outcomes framework 2015/16.

[19] von Essen L (2004). Proxy ratings of patient quality of life: factors related to patient-proxy agreement. Acta Oncol.

[20] Steel JL, Geller DA, Carr BI (2005). Proxy ratings of health related quality of life in patients with hepatocellular carcinoma. Qual Life Res.

[21] Rand S, Caiels J (2015). Using proxies to assess quality of life: a review of the issues and challenges.

[22] Schalock RL, Brown I, Brown R, Cummins RA, Felce D, Matikka L, Keith KD, Parmenter T (2002). Conceptualization, measurement, and application of quality of life for persons with intellectual disabilities: report of an international panel of experts. Ment Retard.

[23] Verdugo MA, Schalock RL, Keith KD, Stancliffe RJ (2005). Quality of life and its measurement: important principles and guidelines. J Intellect Disabil Res.

[24] Caiels J, Rand S, Crowther T, Forder J, Collins G (2016). Developing a proxy-report measure of social care-related quality of life.

[25] Brod M, Tesler L, Christensen T (2009). Qualitative research and content validity: developing best practices based on science and experience. Qual Life Res.

[26] Willis G (2005). Cognitive interviewing: a tool for improving questionnaire design: London: sage.

[27] Beatty PC, Willis G (2007). The practice of cognitive interviewing. Public Opin Q.

[28] Frost MH, Reeve BB, Liepa AM, Stauffer JW, Hays RD (2007). What is sufficient evidence for the reliability and validity of patient-reported outcome measures?. Value Health.

[29] NHS Digital. Personal Social Services Adult Social Care Survey, England - 2014-15, http://content.digital.nhs.uk/catalogue/PUB18642. 2005.

[30] Gil Z, Abergel A, Spektor S, Khafif A, Fliss DM (2004). Patient, caregiver, and surgeon perceptions of quality of life following anterior skull base surgery. Arch Otolaryngol Head Neck Surg.

[31] Becchi A, Rucci P, Placentino A, Neri G, de Girolamo G (2004). Quality of life in patients with schizophrenia--comparison of self-report and proxy assessments. Soc Psychiatry Psychiatr Epidemiol.

[32] Rebollo P, Alvarez-Ude F, Valdes C, Estebanez C (2004). Different evaluations of the health related quality of life in dialysis patients. J Nephrol.

[33] Hung SY, Pickard AS, Witt WP, Lambert BL (2007). Pain and depression in caregivers affected their perception of pain in stroke patients. J Clin Epidemiol.

[34] Schmidt S, Power M, Green A, Lucas-Carrasco R, Eser E, Dragomirecka E, Fleck M (2010). Self and proxy rating of quality of life in adults with intellectual disabilities: results from the DISQOL study. Res Dev Disabil.

[35] Crespo M (2012). Bernaldo de Quiros M, Gomez MM, Hornillos C. Quality of life of nursing home residents with dementia: a comparison of perspectives of residents, family, and staff. Gerontologist.

[36] Gomez-Gallego M, Gomez-Amor J, Gomez-Garcia J (2012). Determinants of quality of life in Alzheimer's disease: perspective of patients, informal caregivers, and professional caregivers. Int Psychogeriatr.

[37] Makai P, Brouwer WB, Koopmanschap MA, Nieboer AP (2012). Capabilities and quality of life in Dutch psycho-geriatric nursing homes: an exploratory study using a proxy version of the ICECAP-O. Qual Life Res.

[38] Graeske J, Fischer T, Kuhlmey A, Wolf-Ostermann K (2012). Quality of life in dementia care: differences in quality of life measurements performed by residents with dementia and by nursing staff. Aging Ment Health.

[39] Pickard AS, Knight SJ (2005). Proxy evaluation of health-related quality of life: a conceptual framework for understanding multiple proxy perspectives. Med Care.

[40] QSR International Ltd. NVivo qualitative data analysis software, Version 10. 2012.

[41] Rand S, Malley J, Netten A (2012). Identifying the impact of Adult social care (IIASC): interim technical report.

[42] Gabbe BJ, Lyons RA, Sutherland AM, Hart MJ, Cameron PA (2012). Level of agreement between patient and proxy responses to the EQ-5D health questionnaire 12 months after injury. J Trauma Acute Care Surg.

[43] Beadle-Brown J, Murphy G, Di TM (2009). Quality of life for the Camberwell cohort. J Appl Res Intellect Disabil.

[44] Schiffczyk C, Jonas C, Lahmeyer C, Muller F, Riepe MW (2011). Gender-dependence of substituted judgment on quality of life in patients with dementia. BMC Neurol.

[45] Muus I, Petzold M, Ringsberg KC (2009). Health-related quality of life after stroke: reliability of proxy responses. Clin Nurs Res.

[46] Edelman P, Fulton BR, Kuhn D (2004). Comparison of dementia-specific quality of life measures in adult day centers. Home Health Care Serv Q.

[47] Sloane PD, Zimmerman S, Williams CS, Reed PS, Gill KS, Preisser JS. Evaluating the quality of life of long-term care residents with dementia. Gerontologist. 2005;45 Spec No 1(1):37-49. 10.1093/geront/45.suppl_1.37 16230748

[48] Jonsson L, Andreasen N, Kilander L, Soininen H, Waldemar G, Nygaard H, Winblad B, Jonhagen ME, Hallikainen M, Wimo A (2006). Patient- and proxy-reported utility in Alzheimer disease using the EuroQoL. Alzheimer Dis Assoc Disord.

[49] Milne DJ, Mulder LL, Beelen HC, Schofield P, Kempen GI, Aranda S (2006). Patients' self-report and family caregivers' perception of quality of life in patients with advanced cancer: how do they compare?. Eur J Cancer Care (Engl).

[50] Naglie G, Tomlinson G, Tansey C, Irvine J, Ritvo P, Black SE, Freedman M, Silberfeld M, Krahn M (2006). Utility-based quality of life measures in Alzheimer's disease. Qual Life Res.

[51] Hoe J, Katona C, Orrell M, Livingston G (2007). Quality of life in dementia: care recipient and caregiver perceptions of quality of life in dementia: the LASER-AD study. Int J Geriatr Psychiatry.

[52] Arlt S, Hornung J, Eichenlaub M, Jahn H, Bullinger M, Petersen C (2008). The patient with dementia, the caregiver and the doctor: cognition, depression and quality of life from three perspectives. Int J Geriatr Psychiatry.

[53] Zimmermann F, Endermann M (2008). Self-proxy agreement and correlates of health-related quality of life in young adults with epilepsy and mild intellectual disabilities. Epilepsy Behav.

[54] Huang HL, Chang MY, Tang JS, Chiu YC, Weng LC (2009). Determinants of the discrepancy in patient- and caregiver-rated quality of life for persons with dementia. J Clin Nurs.

[55] Kunz S (2010). Psychometric properties of the EQ-5D in a study of people with mild to moderate dementia. Qual Life Res.

[56] Schiffczyk C, Romero B, Jonas C, Lahmeyer C, Muller F, Riepe MW (2010). Generic quality of life assessment in dementia patients: a prospective cohort study. BMC Neurol.

[57] Jones JM, McPherson CJ, Zimmermann C, Rodin G, Le LW, Cohen SR (2011). Assessing agreement between terminally ill cancer patients' reports of their quality of life and family caregiver and palliative care physician proxy ratings. J Pain Symptom Manag.

[58] Bruvik FK, Ulstein ID, Ranhoff AH, Engedal K (2012). The quality of life of people with dementia and their family carers. Dement Geriatr Cogn Disord.

[59] Whynes DK, Sprigg N, Selby J, Berge E, Bath PM (2013). Testing for differential item functioning within the EQ-5D. Med Decis Mak.

[60] Claes C, Vandevelde S, Van Hove G, van Loon J, Verschelden G, Schalock R (2012). Relationship between self-report and proxy ratings on assessed personal quality of life-related outcomes. J Policy Pract Intellect Disabil.

[61] Moyle W, Murfield JE, Griffiths SG, Venturato L (2012). Assessing quality of life of older people with dementia: a comparison of quantitative self-report and proxy accounts. J Adv Nurs.

[62] Sheehan BD, Lall R, Stinton C, Mitchell K, Gage H, Holland C, Katz J (2012). Patient and proxy measurement of quality of life among general hospital in-patients with dementia. Aging Ment Health.

[63] Arons AM, Krabbe PF, Scholzel-Dorenbos CJ, van der Wilt GJ, Rikkert MG (2013). Quality of life in dementia: a study on proxy bias. BMC Med Res Methodol.

[64] Yeaman PA, Kim DY, Alexander JL, Ewing H, Kim KY (2013). Relationship of physical and functional independence and perceived quality of life of veteran patients with Alzheimer disease. Am J Hosp Palliat Care.

[65] Zucchella C, Bartolo M, Bernini S, Picascia M, Sinforiani E. Quality of life in alzheimer disease: A comparison of patients' and caregivers' points of view. Alzheimer Dis Assoc Disord. 2015;29(1):50–4. 10.1097/WAD.0000000000000050 24936799

[66] Bryan S, Hardyman W, Bentham P, Buckley A, Laight A (2005). Proxy completion of EQ-5D in patients with dementia. Qual Life Res.

[67] Crespo M, Hornillos C, de Quiros MB (2013). Factors associated with quality of life in dementia patients in long-term care. Int Psychogeriatr.

